# Question framing affects accurate-inaccurate discrimination in responses to sharing questions, but not in responses to accuracy questions

**DOI:** 10.1038/s41598-024-80296-3

**Published:** 2024-11-22

**Authors:** Raoul Bell, Axel Buchner

**Affiliations:** Department of Experimental Psychology, Heinrich Heine University Düsseldorf, Düsseldorf, Germany

**Keywords:** Fake news, Truth judgment, Verbal framing effect, Accuracy prompt, Sharing intention, Psychology, Human behaviour

## Abstract

Previous research suggests that even when people are capable of judging to the best of their knowledge whether claims are accurate or inaccurate, they do not sufficiently discriminate between accurate and inaccurate information when asked to consider whether they would share stories on social media. However, question framing (“To the best of your knowledge…”, “Would you consider…?”) differed between the questions about accuracy and the questions about sharing. Here we examine the effects of question framing on responses to accuracy questions and responses to sharing questions. The framing of accuracy questions had no effect on accurate-inaccurate discrimination. In contrast, accurate-inaccurate discrimination in response to sharing questions increased when participants were asked to respond, to the best of their knowledge, whether they would share claims compared to when they were asked whether they would consider sharing stories. At a theoretical level, the findings support the inattention-based account, according to which contextual cues shifting the focus toward accuracy can enhance accurate-inaccurate discrimination in sharing responses. At a methodological level, these findings suggest that researchers should carefully attend to the verbal framing of questions about sharing information on social media, as the framing may significantly influence participants’ focus on accuracy.

The widespread use of the internet makes it possible to easily access, provide and disseminate information. On the positive side, the efficient flow of information can help people to solve problems, make informed decisions, enhance education and promote democracy by facilitating political participation^1–6^. On the negative side, however, this same ease of sharing information also allows for the spread of inaccurate content to undermine public discourse and individual decision making. One notable example is fake news—inaccurate content presented as authentic news, often intended to deceive internet users to further agendas that may be more or less obvious^7–10^. Democracy relies on citizens being accurately informed and maintaining trust in the political process. Political fake news may lead to manipulated public opinion, swayed elections and polarized societies, thereby weakening democracy^11–13^. Similarly, health-related fake news can mislead people about the severity and nature of health conditions as well as the effectiveness and safety of medical treatments and it can generally erode the trust in science, leading to false beliefs and risky health behaviors such as avoiding vaccinations, using unproven and potentially dangerous treatments or ignoring public health guidelines^14,15^. Given these damaging societal and individual effects, it is crucial to understand why people share inaccurate information on social media. This topic is urgent, as fake news is likely to increase both in quantity and plausibility due to the ongoing advances in generative artificial intelligence (AI)^16^.

Pennycook and colleagues^17,18^ tested three competing accounts of why people share inaccurate information on social media. First, according to the *preference-based account*, people do not care whether the headlines they share are accurate or inaccurate because they prioritize other objectives such as entertaining their audience or promoting their political views, and thus people do not discriminate between accurate and inaccurate information because they are only interested in whether the information furthers their goals. Second, according to the *confusion-based account*, people lack the ability to discriminate between accurate and inaccurate information. Third, according to the *inattention-based account* people generally consider it important to share only accurate information on social media and are able to discriminate between accurate and inaccurate information reasonably well when directly asked to judge the accuracy of the information, but they fail to pay sufficient attention to accuracy when sharing information on social media because they are preoccupied with other concerns such as whether their post will receive engagement or positive reactions.

Pennycook et al.^18^ directly asked their participants to indicate, on a scale ranging from “not at all important” to “extremely important”, how important it was for them to share only accurate information. The modal answer was “extremely important” which, along with accompanying experimental data, provides evidence against the preference-based account. In two highly influential studies with the same innovative basic design, participants in an accuracy condition were asked to decide, to the best of their knowledge, whether the claims in the headlines they were presented with were accurate. Participants in a sharing condition were asked whether they would consider sharing the stories online. Participants were able to discriminate between accurate and inaccurate statements fairly well when they were directly asked to evaluate the accuracy of claims to the best of their knowledge, ruling out the confusion-based account. In contrast, participants were less likely to discriminate between accurate and inaccurate statements when considering whether they would share stories. Pennycook et al.^18^ interpreted these findings as suggesting that people are well able to discriminate between accurate and inaccurate information but may nevertheless fail to sufficiently consider accuracy when sharing information on social media, in line with the inattention-based account [see also^17^].

The inattention-based account promises immediate and practical solutions to the challenge of fighting fake news in that contextual cues that cause people to focus more closely on accuracy should effectively cause social-media users to discriminate more between accurate and inaccurate information in their sharing responses. This short-term perspective is appealing for practitioners because it offers cost-effective solutions and it is also attractive for researchers because the effectiveness of short-term interventions can readily be assessed. It is therefore not surprising that the inattention-based account has proven to be extremely influential in guiding research and discussion on how to combat inaccurate information on social media^19–22^. The account is also supported by findings showing that the discrimination between accurate and inaccurate statements in sharing decisions is affected by situational factors such as accuracy prompts^17,18,23^ and individual factors such as the tendency for cognitive reflection^17,24^.

However, an alternative explanation exists for why participants in the studies of Pennycook et al.^17,18^ discriminated reasonably well between accurate and inaccurate information when responding to the questions about accuracy but were less likely to do so when responding to the questions about sharing information on social media. This explanation is much more prosaic than the inattention-based account and refers to the framing of the questions. Specifically, regarding accuracy, participants were asked “*To the best of your knowledge*, is the *claim* in the above headline accurate?”, whereas, regarding sharing, participants were asked “*Would you consider* sharing this *story* online (for example, through Facebook or Twitter)?” (emphasis added).

Questions such as those just mentioned function as frames^25^ that direct attention to particular aspects of the information relevant to the responses^26,27^. According to classical framing theory, frames provide interpretive structures that guide individuals’ understanding of a situation and set expectations for their behavior^25,28^. In line with this understanding, prompting individuals to “consider” sharing information may activate a framework suggesting a hypothetical, exploratory engagement, where respondents may be less likely to rigorously assess the accuracy of the information. In contrast, the frame “to the best of your knowledge” directly prompts individuals to retrieve relevant knowledge from memory, encouraging them to critically evaluate the accuracy of the information at hand. According to models of decision making, the consideration stage is an initial step in which potential alternative responses are retrieved from memory *before* being deliberated and critically evaluated^29–31^. To illustrate, when seeing a strangely shaped shiny object flying past the window, many people may briefly consider, among other explanations, the unlikely hypothesis that it could be an alien spaceship. However, if pressured to respond to the best of their knowledge about whether the shiny object was actually an alien spaceship, many of those who were willing to consider the alien-spaceship hypothesis may come to the conclusion that the answer is “no”. Additionally, the two framings also differ in the use of the terms “claim” and “story”, as highlighted in the questions above. A “claim” may be associated with a need for accuracy whereas accuracy may not be a primary requirement for a “story”. Thus, the framing of the question alone may explain why participants put more focus on accuracy when judging *claims to the best of their knowledge* than when *considering stories*.

Given the extensive evidence for the influence of verbal framing on behavior^32–37^, it is important to examine whether the framing of the questions is the cause of the dissociation between the responses to the accuracy questions and the responses to the sharing questions. Testing how question framing affects the responses to accuracy and sharing questions is important, not the least because questions with the framing scrutinized here have been used by the original authors in several other studies^23,24,38–42^ as well as by other research groups^22,43–47^. According to the inattention-based account^17,18^, people should be well able to discriminate between accurate and inaccurate statements in response to accuracy questions but should discriminate less between accurate and inaccurate statements in response to sharing questions. If, however, the differences in the framing of the questions were the driving factor for the dissociation, then the observed differences in accurate-inaccurate discrimination between accuracy questions and sharing questions should disappear if the framing is the same for accuracy questions and sharing questions. Specifically, people’s accurate-inaccurate discrimination should be generally better when asked to respond to claims to the best of their knowledge and worse when asked to consider stories, irrespective of whether the questions are about accuracy or sharing. This *question-framing hypothesis* is based on the assumption that asking participants to respond to claims to the best of their knowledge implies a plea to respond based on factual knowledge, which should lead to a focus on accuracy and, hence, to better accurate-inaccurate discrimination. In contrast, asking participants to consider stories plausibly encourages less scrutiny of a statement, resulting in a comparatively lower emphasis on accuracy and, hence, a lower level of accurate-inaccurate discrimination. The question-framing hypothesis thus implies that the previously observed dissociation between sharing responses and accuracy responses^17,18^ is solely due to the difference in the verbal framing of the accuracy questions compared to the sharing questions.

## Experiment 1

### Methods

#### Participants

The participants were recruited by advertising the experiment on the homepage of the Psychology Department, on social media and via email, mostly targeting students of Heinrich Heine University Düsseldorf. We aimed at recruiting as many participants as possible within the three weeks during which the experiment was online. The data of 41 participants who had started to respond to accuracy or sharing questions had to be excluded because these participants did not complete the experiment or withdrew their consent to the use of their data. The data of four participants had to be excluded because these participants were under 18 years old and thus could not legally consent to the use of their data. The final data set comprised the data of *N* = 451 participants (371 female, 76 male, 4 diverse) with a mean age of 24 (*SD* = 8) years, most of whom (415) were native German speakers. Given that participants were mostly recruited among students, the sample was well-educated—98% of the participants had a qualification at the university-entrance level or higher. On average participants reported to spend 3 h (*SD* = 2) daily on social media. When asked to indicate, on a scale from “not at all” to “extremely”, how important it was to share only accurate content on social media, the participants’ modal answer was that they found it “extremely” important. This is parallel to what was reported by Pennycook et al.^18^. When asked to indicate, on a scale from “never” to “all the time”, how often they shared news on social media, the modal response was that they “rarely” did so. Participants were randomly assigned to four groups that differed with respect to the type and framing of the questions about statements participants were presented with. Specifically, 111 participants were asked to decide, to the best of their knowledge, whether the claims in the headlines were accurate, 118 participants were asked whether they would consider that the stories could be accurate, 105 participants were asked to decide, to the best of their knowledge, whether they would share the claims in the headlines online and 117 participants were asked to consider whether they would share the stories online. A sensitivity analysis with G*Power^56^ showed that, with a sample size of *N* = 451 participants, a main effect of question framing (“To the best of your knowledge…”, “Would you consider…”) on the accurate-inaccurate discrimination as small as η_p_^2^ = 0.03 could be detected at an α level of 0.05 with a statistical power of 1 – β = 0.95.

#### Ethics statement

The Ethics Committee of the Faculty of Mathematics and Natural Sciences at Heinrich Heine University Düsseldorf has granted ethical approval for the experiments reported in this article. The experiments were conducted in compliance with the Declaration of Helsinki. Prior to participation, informed content was obtained from all participants. At the end of the experiment, participants were informed that, in addition to accurate statements, inaccurate statements had been presented. They were then shown each inaccurate statement along with its corresponding accurate version. Finally, participants were given the opportunity to withdraw their consent to the use of their data.

#### Materials

**Fig. 1:**
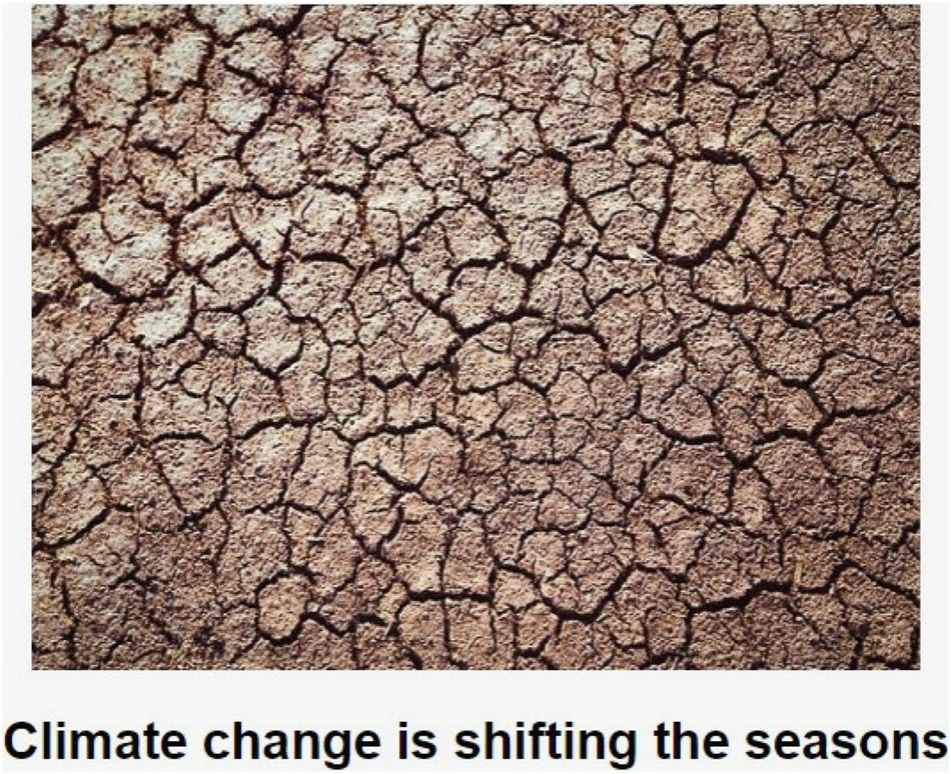
An example statement taken from a fact-checking service provided by the public broadcasting service in Germany^48^. The picture was taken from the Open Affective Standardized Image Set [OASIS^51^]. The statement is the English translation of one of the German statements used in Experiments 1 and 2, which was used in Experiment 3.

A list of 50 accurate and 50 inaccurate statements were taken from a fact-checking service provided by the public broadcasting service in Germany with the intent to combat inaccurate information^48^. In other words, we relied on the fact checkers of Germany’s public broadcasting service to evaluate the truthfulness of the statements. The statements covered a range of topics including medical, environmental and scientific subjects. Examples of accurate statements are “Noise increases the risk of heart attack” and “Offshore wind farms can change marine ecosystems”. Examples of inaccurate statements are “The sudden infant death syndrome is caused by vaccinations” and “Harvard study proves: Wind turbines are partly to blame for global warming”. Each statement was accompanied by a suitable picture to mimic the typical presentation of such statements on social media platforms [cf.^49^]. The pictures accompanying each statement were sourced from internet resources and image databases specifically designed for psychological research^50–52^. An example statement, as presented in the present experiments, is shown in Fig. 1.

Statements for the main experiment were selected based on the outcomes of a separate *norming study* involving *N* = 240 participants, each of whom received a mix of 50 accurate and 50 inaccurate statements in an order that was randomized separately for each individual. Participants were asked to assess the accuracy of each statement. The statements were presented in the same way as in the condition of the experiment proper in which accuracy was to be assessed using the *to-the-best-of-your-knowledge* framing. Participants were asked: “To the best of your knowledge, is the claim in the headline accurate?” with “yes” and “no” as response options. For the experiment proper, we selected the 20 accurate headlines that had received the highest proportion of “yes” responses (*M* = 0.73, *SD* = 0.11) and the 20 inaccurate headlines that had received the lowest proportion of “yes” responses (*M* = 0.23, *SD* = 0.09). These selection criteria ensured that accurate-inaccurate discrimination, given the *to-the-best-of-your-knowledge* framing, was closely aligned with that observed in studies upon which the present research was based^17,18^.

#### Procedure

At the start of the experiment proper, participants were instructed to complete the task independently and without interruptions. If this was not possible, they were advised to exit the experiment by closing the browser window. After having consented to participate, participants were informed, depending on the framing of the questions in the condition they were assigned to, that they would see a series of headlines or stories taken from social media such as Facebook, X (formerly Twitter), WhatsApp, Telegram, Signal or Instagram. When translating the questions to German for the German-speaking participants, care was taken to ensure that the wordings of the questions was as close as possible to those used in previous studies^17,18^. The statements were presented individually, one after another and in different random order for each participant, in the format illustrated in Fig. 1. Translated into English, the accuracy questions were “To the best of your knowledge, is the claim in the headline accurate?” and “Would you consider that the story could be accurate?”. The sharing questions were “To the best of your knowledge, would you share the claim in the headline online?” and “Would you consider sharing the story online?”. Participants answered the questions by selecting a “yes” or “no” checkbox. Which checkbox was presented as the left checkbox was counterbalanced across participants. After having selected a checkbox, a “continue” button was presented. When this button was clicked, the next question was presented.

When participants had responded to all questions, they were transferred to a page on which they were informed that some of the statements they had just seen had been inaccurate. They were told that the accurate statement would be provided in the next section of the experiment. Following current scientific recommendations on how to effectively correct misinformation^53^, each inaccurate statement was first repeated before it was refuted. For instance, participants were informed: “*The following is inaccurate*: Sudden infant death syndrome is caused by vaccinations”. Each inaccurate statement was immediately followed by a detailed correction contrasting the inaccurate statement with accurate information. For instance, the correction to the statement just mentioned was: “*The following is correct*: Cases of sudden infant death syndrome have been falling steadily for years, although the number of vaccinations has remained at least the same. Studies show that the risk of sudden infant death is not increased after vaccinations. Berlin scientists found that the opposite is true: As vaccination rates increase, cases of sudden infant death syndrome fall.” The corrections were taken from the same fact-checking service as the accurate and inaccurate statements^48^.

At the conclusion of the experiment, participants were asked to indicate how often they shared news on social media using a 5-point scale (1 = “never”, 2 = “rarely”, 3 = “sometimes”, 4 = “frequently”, 5 = “constantly”). They were also asked to rate the importance of sharing only accurate content on social media, using another 5-point scale (1 = “not at all”, 2 = “a little”, 3 = “medium”, 4 = “very”, 5 = “extremely”). Furthermore, participants were asked how much time (in hours per day) they spent on social media (for example, Facebook, X, WhatsApp, Telegram, Signal or Instagram). After having reported their age, gender, native language and education level, participants were informed about the purpose of the study. They were then given the option to withdraw their consent to the use of their data—which they had given at the start of the experiment—by selecting the “No, I withdraw my consent to the use of my data” option. Finally, participants were thanked for their participation.

### Results

**Fig. 2:**
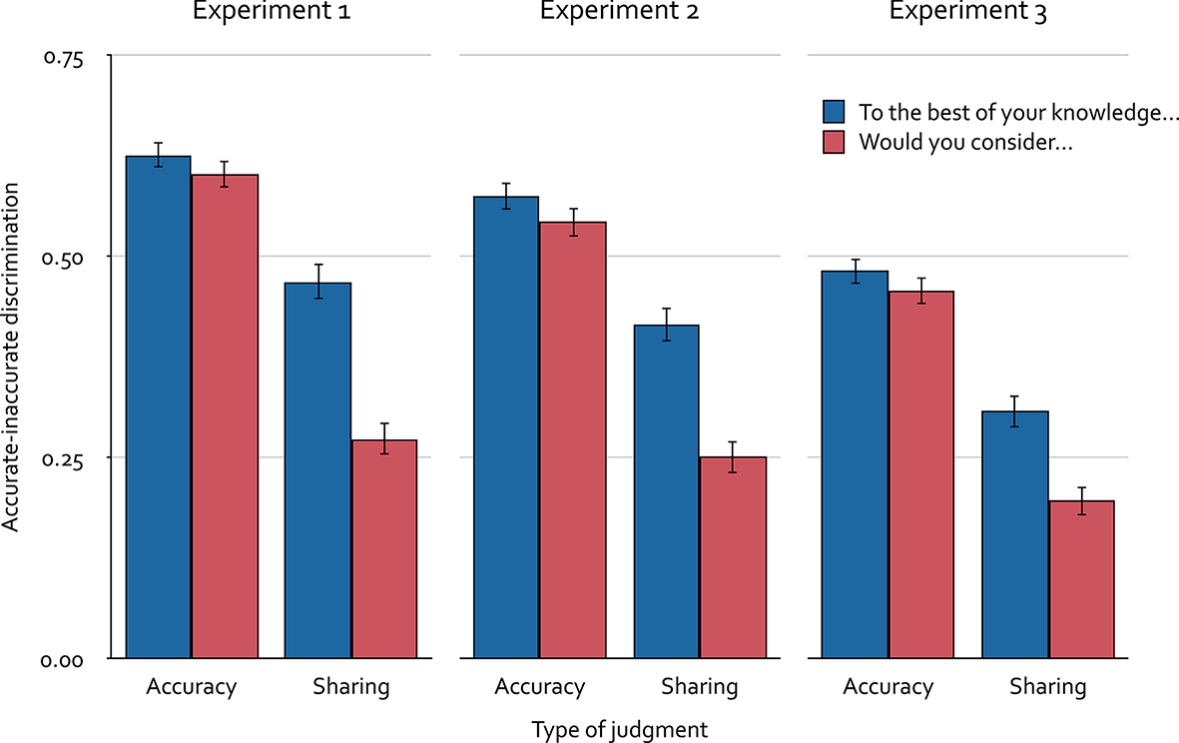
Mean accurate-inaccurate discrimination index *P*_r_^54^ as a function of type of judgment (accuracy, sharing) and question framing (“To the best of your knowledge…”, “Would you consider…?”) in Experiments 1, 2 and 3. Error bars represent the standard errors of the means.

The significance level was set to α = 0.05 for all analyses reported in this article. As the main hypotheses pertain to participants’ accurate-inaccurate discrimination, the discrimination index *P*_r_ of the two-high threshold model was calculated for each individual based on the proportions of “yes” responses to accurate and inaccurate statements. This discrimination measure was selected because it has been favorably evaluated in a series of validation experiments for the present 2 × 2 data structure compared to competing measurement models^54^. The mean discrimination index *P*_r_ is displayed in Fig. 2. For completeness, the mean bias index *B*_r_, indicating the tendency toward responding “yes” when in an uncertain state, is reported in Table 1.

A 2 × 2 between-subjects analysis of variance was performed to examine how type of judgment (accuracy, sharing) and question framing (“To the best of your knowledge…”, “Would you consider…”) influenced accurate-inaccurate discrimination, as measured by the discrimination index *P*_r_. Accurate-inaccurate discrimination was significantly better when responding to accuracy questions than when responding to sharing questions, *F*(1, 447) = 169.32, *p* < 0.001, η_p_^2^ = 0.27. Additionally, the framing “To the best of your knowledge….” on average led to better accurate-inaccurate discrimination than the framing “Would you consider…”, *F*(1, 447) = 34.38, *p* < 0.001, η_p_^2^ = 0.07. Importantly, there was a significant interaction between type of judgment and question framing, *F*(1, 447) = 20.96, *p* < 0.001, η_p_^2^ = 0.04. The way the accuracy questions were framed did not influence accurate-inaccurate discrimination, *t*(227) = 1.06, *p* = 0.29, *d* = 0.14. In contrast, accurate-inaccurate discrimination was better when the framing of the sharing questions was “To the best of your knowledge…” than when it was “Would you consider…”, *t*(220) = 6.51, *p* < 0.001, *d* = 0.88. The results thus suggest that question framing has no influence on accurate-inaccurate discrimination when responding to accuracy questions but affects accurate-inaccurate discrimination when responding to sharing questions.

**Table 1:** Mean bias index *B*_r_measuring the tendency toward responding “yes” when in an uncertain state^54^, as a function of type of judgment (accuracy, sharing) and question framing (“To the best of your knowledge…”, “Would you consider…”). Values in parentheses represent the standard errors of the means.

	Accuracy				Sharing			
	“To the best of your knowledge…“		“Would you consider…”		“To the best of your knowledge…“		“Would you consider…”	
	Mean	(Standard error)	Mean	(Standard error)	Mean	(Standard error)	Mean	(Standard error)
Experiment 1	0.41	(0.02)	0.59	(0.02)	0.19	(0.02)	0.15	(0.02)
Experiment 2	0.37	(0.02)	0.49	(0.02)	0.32	(0.02)	0.26	(0.02)
Experiment 3	0.37	(0.02)	0.37	(0.02)	0.23	(0.02)	0.22	(0.02)

### Discussion

The framing of questions did not affect accurate-inaccurate discrimination uniformly but instead had distinct effects depending of whether participants responded to questions about accuracy or to questions about sharing. In response to accuracy questions, accurate-inaccurate discrimination was equally good regardless of how the questions were framed. In response to the sharing questions, in contrast, the requirement to respond to claims to the best of their knowledge rather than to consider stories caused participants to discriminate better between accurate and inaccurate information.

## Experiment 2

A limitation of Experiment 1 is that, even though the experiment was conducted online, the participants were mostly students. Students may differ in how they respond to the accuracy and sharing questions from participants recruited via online panels which were primarily focused on in previous research^17,18^. Specifically, it seems conceivable that students are particularly responsive to verbal framing effects, for example, because they are often exposed to nuanced language in academic settings, which could heighten their sensitivity to how information is presented. For this reason and because direct replications are generally important^55^, Experiment 2 was designed to serve as a direct replication of Experiment 1, with the main difference that the participants in Experiment 2 were recruited through an online panel provider.

### Methods

#### Participants

To recruit a sample of active online users with residency in Germany, participants were recruited via the online panel provider *Cint* (https://www.cint.com/*).* We aimed at recruiting about 600 participants and ended data collection at the end of the day this criterion was reached. The data of 25 participants who had started to respond to accuracy or sharing questions had to be excluded because these participants did not complete the experiment or withdrew their consent to the use of their data. The data of two participants had to be excluded because these participants were under 18 years old and thus could not legally consent to the use of their data. The final data set comprised the data of *N* = 595 participants (323 female, 268 male, 4 diverse) with a mean age of 47 (*SD* = 16) years, most of whom (567) were native German speakers. The sample was characterized by a diverse range of education. As an indication, 54% (compared to 98% in Experiment 1) had a qualification at the university-entrance level or higher. When asked to indicate, on a scale from “not at all” to “extremely”, how important it was to share only accurate content on social media, the participants’ modal answer was that they found it “extremely” important. This is again parallel to what was reported by Pennycook et al.^18^. When asked to indicate, on a scale from “never” to “all the time”, how often they shared news on social media, the modal response was that they “rarely” did so. Participants were randomly assigned to four groups that differed with respect to the type and framing of the questions about statements participants were presented with. Specifically, 142 participants were asked to decide, to the best of their knowledge, whether the claims in the headlines were accurate, 148 participants were asked whether they would consider that the stories could be accurate, 154 participants were asked to decide, to the best of their knowledge, whether they would share the claims in the headlines online and 151 participants were asked to consider whether they would share the stories online. A sensitivity analysis with G*Power^56^ showed that, with a sample size of *N* = 595 participants, a main effect of question framing (“To the best of your knowledge…”, “Would you consider…”) on accurate-inaccurate discrimination as small as η_p_^2^ = 0.02 could be detected at an α level of 0.05 with a statistical power of 1 – β = 0.95.

#### Materials and procedure

Materials and procedure were identical to those of Experiment 1 except that the question about daily social media usage was omitted.

### Results

The mean discrimination index *P*_r_, representing accurate-inaccurate discrimination, is displayed in Fig. 2. For completeness, the mean bias index *B*_r_, indicating the tendency toward responding “yes” when in an uncertain state^54^, is reported in Table 1.

A 2 × 2 between-subjects analysis of variance was performed to examine how type of judgment (accuracy, sharing) and question framing (“To the best of your knowledge…”, “Would you consider…”) influenced accurate-inaccurate discrimination, as measured by the discrimination index *P*_r_. Accurate-inaccurate discrimination was significantly better when responding to accuracy questions than when responding to sharing questions, *F*(1, 591) = 139.81, *p* < 0.001, η_p_^2^ = 0.19. Additionally, the framing “To the best of your knowledge….” on average led to better accurate-inaccurate discrimination than the framing “Would you consider…”, *F*(1, 591) = 26.43, *p* < 0.001, η_p_^2^ = 0.04. Importantly, there was a significant interaction between type of judgment and question framing, *F*(1, 591) = 11.97, *p* < 0.001, η_p_^2^ = 0.02. The way the accuracy questions were framed did not influence accurate-inaccurate discrimination, *t*(288) = 1.36, *p* = 0.177, *d* = 0.16. In contrast, accurate-inaccurate discrimination was better when the framing of the sharing questions was “To the best of your knowledge…” compared to when it was “Would you consider…”, *t*(303) = 5.54, *p* < 0.001, *d* = 0.63. The results thus confirm that question framing has no influence on accurate-inaccurate discrimination when responding to accuracy questions but affects accurate-inaccurate discrimination when responding to sharing questions.

### Discussion

Even though, in Experiment 2, we used a panel provider to recruit participants with a more diverse educational background than that of the participants of Experiment 1 who were mostly students, the results were strikingly similar to those of Experiment 1. As in Experiment 1, the hypothesis that question framing affects accurate-inaccurate discrimination in response to accuracy questions has to be rejected. By contrast, accurate-inaccurate discrimination in response to sharing questions is increased when participants are asked to respond to claims to the best of their knowledge rather than to consider stories.

## Experiment 3

A limitation of Experiments 1 and 2 is that both experiments were conducted in German with German participants. Therefore, the results may have depended on nuances of translation. This issue is more serious than it might appear at first glance. This is so because a word-by-word translation of the German phrase for “to the best of your knowledge” would be “according to the best of one’s knowledge and conscience”. The German translation thus refers not only to knowledge but also to conscience which may be conceived of as a considerable intensification of the request expressed by the phrase. To test whether the framing effects observed in Experiments 1 and 2 were due to the peculiarity of how “to the best of your knowledge” is translated into German, we performed a conceptual replication of Experiments 1 and 2 with the main difference that the experiment was conducted in English using a phrasing of the questions as in the studies of Pennycook et al.^17,18^ and relying on an online panel provider to recruit participants residing in Great Britain.

### Methods

#### Participants

To collect data from a sample of active online users with residency in Great Britain, participants were recruited via the online panel provider *Bilendi* (https://www.bilendi.de/). We aimed at recruiting about 600 participants and ended data collection at the end of the day this criterion was reached. The data of 53 participants who had started to respond to accuracy or sharing questions had to be excluded because these participants did not complete the experiment or withdrew their consent to the use of their data. The data of one participant had to be excluded because this participant was under 18 years old and thus could not legally consent to the use of their data. The final data set comprised the data of *N* = 610 participants (291 female, 318 male, 1 diverse) with a mean age of 57 (*SD* = 17) years, most of whom (580) were native English speakers. The sample was characterized by a diverse range of education. As an indication, 52% had at least a qualification corresponding to a university entrance qualification in Germany which is roughly comparable to the same figure obtained for the German online-panel sample in Experiment 2. When asked to indicate, on a scale ranging from “not at all” to “extremely”, how important it was to share only accurate content on social media, the participants’ modal answer was that they found it “extremely” important. This is again parallel to what was reported by Pennycook et al.^18^. When asked to indicate, on a scale from “never” to “all the time”, how often they shared news on social media, the modal response was that they “rarely” did so. Participants were randomly assigned to four groups that differed with respect to the type and framing of the questions about statements participants were presented with. Specifically, 155 participants were asked to decide, to the best of their knowledge, whether the claims in the headlines were accurate, 155 participants were asked whether they would consider the stories to be accurate, 150 participants were asked to decide, to the best of their knowledge, whether they would share the claims in the headlines online and 150 participants were asked to consider whether they would share the stories online. A sensitivity analysis with G*Power^56^ showed that, with a sample size of *N* = 610 participants, a main effect of question framing (“To the best of your knowledge…”, “Would you consider…”) on accurate-inaccurate discrimination as small as η_p_^2^ = 0.02 could be detected at an α level of 0.05 with a statistical power of 1 – β = 0.95.

#### Materials and procedure

Materials and procedure were identical to those of Experiment 2, except that the experiment was conducted in English. Depending on the framing condition, the accuracy questions were “To the best of your knowledge, is the claim in the headline accurate?” or “Would you consider the story to be accurate?”; the sharing questions were “To the best of your knowledge, would you share the claim in the headline online?” or “Would you consider sharing the story online?”. This ensured that the questions “To the best of your knowledge, is the claim in the headline accurate?” and “Would you consider sharing the story online?“ corresponded to those used in previous studies^17,18^. Minor modifications were made to the statements to adapt the stimulus material for participants living in Great Britain. For instance, the speed limit mentioned in one of the statements was changed from 30 kilometers per hour to 20 miles per hour.

### Results

The mean discrimination index *P*_r_, representing accurate-inaccurate discrimination, is displayed in Fig. 2. For completeness, the mean bias index *B*_r_, indicating the tendency toward responding “yes” when in an uncertain state^54^, is reported in Table 1.

A 2 × 2 between-subjects analysis of variance was performed to examine how type of judgment (accuracy, sharing) and question framing (“To the best of your knowledge…”, “Would you consider…”) influenced accurate-inaccurate discrimination, as measured by the discrimination index *P*_r_. Accurate-inaccurate discrimination was significantly better when responding to accuracy questions than when responding to sharing questions, *F*(1, 606) = 155.58, *p* < 0.001, η_p_^2^ = 0.20. Additionally, the framing “To the best of your knowledge….” on average led to better accurate-inaccurate discrimination than the framing “Would you consider…”, *F*(1, 606) = 15.39, *p* < 0.001, η_p_^2^ = 0.02. Importantly, there was a significant interaction between type of judgment and question framing, *F*(1, 606) = 6.32, *p* = 0.012, η_p_^2^ = 0.01. The way the accuracy questions were framed did not influence accurate-inaccurate discrimination, *t*(308) = 1.11, *p* = 0.269, *d* = 0.13. In contrast, accurate-inaccurate discrimination was better when the framing of the sharing questions was “To the best of your knowledge…” than when it was “Would you consider…”, *t*(298) = 4.15, *p* < 0.001, *d* = 0.48. The results thus confirm again that question framing has no influence on accurate-inaccurate discrimination when responding to accuracy questions but affects accurate-inaccurate discrimination when responding to sharing questions.

### Discussion

When the instructions and questions are presented in their original English phrasing, parallel to those used by Pennycook et al.^17,18^, the conclusions remain consistent with those of the previous two experiments. The hypothesis that question framing affects accurate-inaccurate discrimination in response to accuracy questions has to be rejected. In contrast, when responding to sharing questions, accurate-inaccurate discrimination is better when participants are required to respond to claims to the best of their knowledge rather than when they are required to consider stories. Therefore, the findings of Experiments 1 and 2 cannot be attributed to peculiarities in the translation of the phrase “to the best of your knowledge” into German.

## General discussion

Understanding why people fail to discriminate between accurate and inaccurate information on social media is crucial for developing effective strategies to combat fake news^7,8^. According to the influential inattention-based account^9^, people fail to sufficiently discriminate between accurate and inaccurate information in their sharing responses even though they find it important to share only accurate information and can, in principle, discriminate fairly well between accurate and inaccurate information when directly asked to judge the accuracy of the information. The reduction in accurate-inaccurate discrimination in response to sharing questions relative to accuracy questions is attributed to a lack of attention: People do not spontaneously pay enough attention to accuracy when sharing information on social media. A foundational finding supporting this account is that people show a lower degree of accurate-inaccurate discrimination when asked whether they would consider sharing stories than when asked to judge the accuracy of claims to the best of their knowledge. However, in previous studies^17,18^ the framing for sharing questions differed systematically from the framing for accuracy questions. Therefore, we examined, in three experiments, how the framing of the questions affects accuracy and sharing responses.

According to the question-framing hypothesis, question framing should affect accuracy and sharing responses in a parallel manner. Specifically, the framing “*To the best of your knowledge*…” should cause participants to focus more on accuracy than the framing “*Would you consider* …” which should be less likely to encourage people to scrutinize the veracity of statements, resulting in a decreased emphasis on accuracy. Importantly, according to the question-framing hypothesis this framing effect should occur for both accuracy questions and sharing questions. Contradicting this prediction implied by the question-framing hypothesis, the results obtained in the present series of experiments demonstrate that question framing did not affect accurate-inaccurate discrimination uniformly but instead had distinct effects depending of whether participants responded to questions about accuracy or to questions about sharing. Question framing did not affect accurate-inaccurate discrimination in response to accuracy questions. In sharp contrast, question framing consistently affected accurate-inaccurate discrimination in response to sharing questions. Specifically, accurate-inaccurate discrimination in response to sharing questions was better when the framing stimulated participants to respond to claims to the best of their knowledge than when they were asked to consider stories. This pattern of results was consistently observed across all three experiments, regardless of whether participants were recruited among university students (Experiment 1) or through online panels in Germany (Experiment 2) or in Great Britain (Experiment 3).

Why does question framing affect accurate-inaccurate discrimination in response to sharing questions but not in response to accuracy questions? Responses to sharing questions may be more susceptible to framing than responses to accuracy questions because sharing on social media may involve multiple goals such as providing accurate content, seeking social approval, fostering social engagement and capturing attention. The competition among multiple goals, only one of which is to share accurate information, may explain why responses to sharing questions are more easily influenced by contextual cues than responses to accuracy questions, where the content of the question itself implies that accuracy is the singular goal. This interpretation is in fact fully consistent with Pennycook et al.’s^17,18^ inattention-based account. An important implication of the inattention-based account is that people fail to discriminate between accurate and inaccurate information in their sharing responses not because they lack the motivation or ability to do so but because they fail to sufficiently attend to accuracy, becoming distracted by competing goals such as that of fostering social engagement. Therefore, contextual cues that prompt people to focus more on accuracy should enhance accurate-inaccurate discrimination in sharing responses. As a practical consequence, any contextual cue that directs the focus of attention toward accuracy can be expected to improve the quality of information shared on social media. Here, asking participants to respond to whether they would share claims on social media to the best of their knowledge rather than to consider sharing stories is such a contextual cue that prompts people to focus more on accuracy. Consequently, accurate-inaccurate discrimination is better when the framing of the sharing questions appeals to people’s knowledge about facts than when it does not, consistent with the assumptions of the inattention-based account. When responding to accuracy questions, the focus is naturally on accuracy, as this is the subject of these questions, even without additional cues to emphasize accuracy. Therefore, asking people to judge the accuracy of claims to the best of their knowledge yields similar outcomes to asking them to consider the accuracy of stories, as both approaches encourage a focus on accuracy.

The present findings fit nicely with those of Capraro and Celadin^21^ who have already shown that the verbal framing of the response options of questions about sharing affects the proportions of accurate and inaccurate statements that are shared. Specifically, when the response option to the question “If you were to see the above article on Facebook, would you consider sharing it?” was phrased as “Yes; I think this news is accurate” instead of only “Yes”, then accurate-inaccurate discrimination in people’s sharing responses was increased. The results of the present study extend these previous findings by showing that subtle changes in the verbal framing of questions cause people to better discriminate between accurate and inaccurate information when deciding what to share on social media. These findings should encourage further investigation into how nuanced framing manipulations can enhance accurate-inaccurate discrimination in sharing decisions on social media. For instance, platforms could ask users “Are you confident that this information is accurate?” rather than simply offering a share button, leveraging framing to reduce the spread of misinformation.

However, as a limitation of the prospect to decrease reliance on fake news through framing interventions, it must be acknowledged that, given the ongoing advances in generative AI^16^, interventions aimed at shifting people’s focus on accuracy may eventually lose their effectiveness. These interventions fundamentally rely on people’s ability to distinguish between accurate and inaccurate information, but as AI-generated misinformation becomes increasingly difficult to discriminate from accurate information, features that prompt people to focus on accuracy will likely become less and less effective. For the time being, however, stimulating people to focus on accuracy may still serve as a useful tool in combating misinformation.

As another limitation of the present study, it must be noted that the question-framing manipulation used here comprised not only the phrases “To the best of your knowledge” and “Would you consider…” but also the terms “claim” and “story”, respectively. As mentioned in the introduction, a “claim” may be associated with a need for accuracy whereas accuracy may not be a primary requirement for a “story”. As a result, we only examined the joint effects of both elements, which implies that the effect of the verbal framing of the questions about sharing on accurate-inaccurate discrimination could be due either to one of these elements or to both elements. Whereas the distinction between these two elements of the framing was not the focus of the present study, future research could explore these nuances further by dissecting the effect of the individual elements of the framing on accurate-inaccurate discrimination.

To summarize, the present study shows that question framing has distinct effects on how well people discriminate between accurate and inaccurate headlines in response to accuracy questions and sharing questions. While the framing of questions does not affect the participants’ accurate-inaccurate discrimination in response to questions about accuracy, asking participants to respond to claims to the best of their knowledge instead of considering stories increases the accurate-inaccurate discrimination in responses to questions about the sharing on social media. At a theoretical level, the present results support the inattention-based account^9^. According to this account, people fail to discriminate between accurate and inaccurate information on social media because they do not pay sufficient attention to accuracy. Asking participants to respond to claims to the best of their knowledge, rather than to consider stories, can be interpreted as a contextual cue that shifts the focus toward accuracy, thereby enhancing accurate-inaccurate discrimination in sharing responses. At a methodological level, these findings indicate that while the phrasing of questions about accuracy may not affect the results, researchers should carefully attend to the questions about sharing on social media, as the verbal framing of such questions may significantly influence the participants’ focus on accuracy. To align the decision-making process with how it occurs on social media, it may be advisable to straightforwardly ask participants, “Do you want to share this information?” or to design experiments that closely mirror the social-media interface [cf.^21,57^].

## Data Availability

The data of the experiments are available at: https://osf.io/xc4wf/.
